# Utilization of bottle gourd (*Lagenaria siceraria* (Mol.) Standl.) pomace for the preparation of instant *kheer* (dessert) mix

**DOI:** 10.1016/j.heliyon.2023.e18533

**Published:** 2023-07-29

**Authors:** Ghan Shyam Abrol, Amit Kumar Singh, Ranjit Pal, Ashwani Kumar, Priyanka Sharma, Gaurav Sharma

**Affiliations:** a Department of Postharvest Technology, College of Horticulture and Forestry, Rani Lakshmi Bai Central Agricultural University, Jhansi, India; b Department of Postharvest Technology, Banda University of Agriculture and Technology, Banda, India; c Department of Fruit Science, College of Horticulture and Forestry, Rani Lakshmi Bai Central Agricultural University, Jhansi, India; d Department of Floriculture and Land Scaping, College of Horticulture and Forestry, Rani Lakshmi Bai Central Agricultural University, Jhansi, India

**Keywords:** Bottle gourd, Pomace, Central composite design, Canonical correlation analysis, Principal component analysis, *Kheer* mix

## Abstract

Bottle gourd pomace, a waste from vegetable processing industry was used to prepare instant *kheer* (dessert) mix. In this study, the bottle gourd was procured from the farm, washed, grated, steam blanched and the grits were further divided into two parts. One part of grits was dried without juice extraction (BGFD- Bottle gourd fresh dried), while, the other half (BGPD- Bottle gourd pomace dried) was dehydrated after extraction of juice. The dehydrated grits were used for the preparation of *kheer* mix and the recipe was optimized using RSM Central Composite Design (CCD). The variables were BGFD and BGPD ranged 3–7 g. The other ingredients with the fixed quantities were milk powder (50 g), sugar (15 g), and small cardamom (1 g). The product was selected based on sensory responses like taste, colour, flavour, texture, and overall acceptability (OAA). The software suggested a *kheer* mix prepared using 7 g BGFD and 3 g BGPD will produce the best sensory scores. The prepared *kheer* mix had a moisture, TSS, carbohydrates, reducing sugars, total sugars, titratable acidity, crude protein, and crude fat content of 7.9%, 27 °B, 72.21%, 10.79%, 16.75%, 0.896% CA, 10.76%, and 7.63%, respectively. The product was rich in energy (400.55 kcal/100 g), total phenols (4.99 mg/100 g), and exhibited strong antioxidant activity (46%). The total plate count on the product on nutrient agar medium was 4.3 × 10^6^ CFU/g. The *kheer* could be prepared by adding 140 mL of water to 70 g of water to kheer mix and cooking it for 10 min. Further, to see the credibility and obtain more clearer patterns, the Canonical Correlation Analysis (CCA) and Principal Component Analysis (PCA) were applied. The overall variation of the BGFD and BGPD on the sensory parameters based on canonical correlation analysis was 92.5%. The sum of Principal Components PC1 and PC2 explained a very high variability (98.2%) among the studied treatments.

## Graphical abstract

**Figure:**
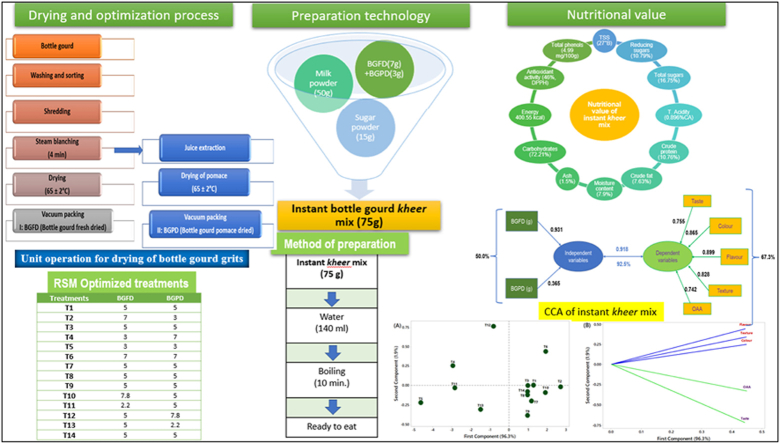

## 1 Introduction

Ready-to-cook or instant food products have become increasingly popular in today's fast-paced world due to their time efficiency and ease of preparation [1]. The concept of convenience food has gained traction in both developed and developing countries, attracting consumers with their attractive packaging, reasonable price, and easy-to-cook ability [[2], [3], [4]]. Additionally, the market for instant food products in India has been rapidly growing, with a projected CAGR rate of over 16% from 2017 to 2023 [5]. The traditional cereal-based instant products, such as bakery food, breakfast cereals, biscuits, noodles, and instant snacks, dominate the market due to their shelf stability, nutritionally enhanced nature, and ease of carrying [6]. However, still there is a need to develop innovative recipes to meet the growing demand for new products. Moreover, with the global population estimated to reach 9.8 billion by 2050, and a projected increase in food demand of 50–60%, there is a need to reduce food waste and utilize all available resources [7]. Approximately one-third of fruit and vegetable production is lost each year, resulting in 1.3 billion tons of waste [8]. Many scientists have shown the nutritional, antioxidant, and antimicrobial properties of fruit and vegetable waste, which can be utilized for various purposes [[9], [10], [11], [12]]. Plant and milk based composite food/drinks are also in trend [[13], [14], [15]] Further, these are ideal for instant product development. Similarly, the pomace left after juice extraction from the fruits and vegetables industry is a good source of fibers, phytochemicals and minerals [16]. The bottle gourd *Lagenaria siceraria* (Mol.) Standl. is an important vegetable of the *Cucurbitaceae* family and commonly known as *Lauki*, Ghia, *Calabash* or Dudhi in India. The edible index of the fruit is 94.17% [17] and is a good source of dietary fibers (0.6%), carbohydrates (2.5%), fat (0.1%), amino acids (leucines 0.8; phenylalanine 0.9; valine 0.3; tyrosine 0.4; alanine 0.5; threonine 0.2; glutamic acid 0.3; serine 0.6; aspartic acid 1.9; cystine 0.6 and cysteine 0.3), mineral (0.5%), vitamin B complex and vitamin C [18,19]. The bottle gourd alone or in combination with other crops especially medicinal crops is used to treat various diseases *viz* diabetes, jaundice colitis, ulcer, piles, hypertension, insanity, congestive cardiac failure and to treat skin diseases [20]. The pulp of the fruit and its extract is used as a sedative, emetic, cooling, purgative, diuretic, pectoral, antibilious, and as a remedy against antimicrobial infections [21,22]. In India during 2017-18, the organsied sector of processing which is 2.26%, processed 60.6 thousand tonnes of bottle gourd and generated around 25.5 thousand tonnes of bottle gourd pomace [23]. Therefore, to utilize this huge waste, in this study, an instant dessert product is prepared using bottle gourd pomace in combination with dried shreds of bottle gourd without juice extraction. The recipe is optimized using Response Surface Methodology (RSM) CCD. Further, Canonical Correlation Analysis (CCA) and Principal Correlation Analysis (PCA) are applied to analyze the data. This study aims to add value to the bottle gourd pomace and develop a new instant dessert product that is both nutritious and appealing to consumers.

## 2 Material and methods

### 2.1 Raw material

Bottle gourds were procured from the Experimental Farm of Vegetable Science, RLBCAU, Jhansi, while, the table sugar, milk powder, small cardamom and packaging materials were purchased from the local market.

### 2.2 Juice extraction

The juice was extracted by pressing from peeled and shredded bottle gourds The remaining biomass was then steam blanched for 4 min and dehydrated in a mechanical dehydrator at 65 ± 2 °C for further experimentation. Although in the previous experiment the whole dried biomass was used to make an instant dessert (*kheer* mix) but it received low sensory scores (data not presented). Consequently, a new experiment was devised and conducted, in which combinations of fresh dried Bottle gourd (BGFD- Bottle gourd fresh dried) and dried pomace left over after juice extraction (BGPD- Bottle gourd pomace dried) were tested using a CCD RSM design.

### 2.3 Optimization process

The RSM Central Composite Design (CCD) with 6 center points and *α*-1.41 was kept to optimize instant dessert from bottle gourd for wider sensory acceptability. The experiment involved a total of 14 runs, and 50 g of milk powder, 15 g of sugar, and 1 g of small cardamom were kept constant for all runs. Taste, colour, flavour, texture, and overall acceptability (OAA) were the different responses assessed during optimization. To determine the best-fit model for each parameter, Fisher's test was applied. The coded equation was used to predict responses based on the given levels of each factor. The high levels of the factors were coded as +1, while the low levels were coded as −1. Comparing the factor coefficients aided in identifying the relative impact of each factor. Following analysis, the software (Design Expert, 13) suggested the best combination, which was then prepared and compared with the experimental values to determine the desirability.

### 2.4 Physico-chemical analysis

#### 2.4.1 Total soluble solids (TSS)

An Erma hand refractometer was utilized to measure the total soluble solid (TSS) contents of the mix. A few drops of the rehydrated mix were squeezed onto the refractometer's prism, and the readings were observed through the eyepiece. The obtained readings were corrected for temperature variation using the International Temperature Correction Table at 20 °C and expressed as °Brix [24].

#### 2.4.2 Reducing and total sugars

A 25-g sample was taken in a 250 mL volumetric flask and mixed with 100 mL of water. The resulting solution was neutralized using 1 N NaOH, and 2 mL of 45% lead acetate was added to it. The mixture was then left for 10 min to settle. Excess lead acetate was removed from the sample by adding 2 mL of 22% potassium oxalate in a 250 mL volumetric flask. The solution was then diluted up to the mark, filtered, and the clear filtrate was used to estimate reducing sugars. The estimation was done by titrating the filtrate against a known quantity of Fehling's A and Fehling's B solution, using methylene blue as an indicator [25]. The percentage of reducing sugars was calculated as follows:

$$Reducing\;sugars\;\left(\%\right)=\frac{Factor\times Dilution}{Titre\;value\times Weight\;of\;sample\;taken}\times 100$$

The total sugars were estimated by adding 5 g of citric acid to a 50 mL calibrated sample solution and heating it for 10 min. For complete inversion of sugars, neutralizing with NaOH and making volume 250 mL in a volumetric flask was done. The total sugars were estimated as *per cent* and calculated as given:

$$Total\;sugars\;as\;invert\;sugars\;\left(\%\right)=\frac{Factor\times Dilution}{Titre\times Weight\;of\;sample\;taken}\times 100$$

%Sucrose=(%totalinvertsugars−%reducingsugars)×0.95%Totalsugars=(%reducingsugars+%sucrose)

#### 2.4.3 Titratable acidity

To determine the titratable acidity, a 5-g sample was taken in a 100 mL volumetric flask and made up to volume by adding distilled water. The solution was filtered, and 10 mL of the filtrate was transferred to a separate conical flask. The 10 mL filtrate was then titrated against 0.1 N sodium hydroxide using phenolphthalein as an indicator. The endpoint was determined by the appearance of a faint pink colour. The titratable acidity was calculated and expressed as a percentage of citric acid according to the method described by Ranganna [26].

#### 2.4.4 Crude protein

The Kjeldahl method was employed to determine the crude protein content. In this method, the amount of reduced nitrogen in the solution is determined by estimating the nitrogen content, which is then titrated with a standard acid. The various nitrogenous compounds present in the sample are converted into ammonium sulphate by boiling with concentrated sulphuric acid. The resulting ammonium sulphate is then decomposed with an alkali (sodium hydroxide), and the liberated ammonia is absorbed in an excess of neutral boric acid. To begin, a sample of 0.5 g weight was taken along with 0.5 g digestion mixture (consisting of 2.5 g SeO_2_ + 20 g CuSO_4_·5H_2_O + 100 g K_2_SO_4_), and this mixture was digested in 25 mL concentrated H_2_SO_4_ for 5 h or until the solution became colourless. The digestion flasks were then allowed to cool overnight at room temperature. The digest was subsequently transferred to a 100 mL volumetric flask and made up to volume with distilled water [26].

Crude Protein (%) = % nitrogen × 6.25.

#### 2.4.5 Crude fat

The Soxtec oil extraction method was utilized to determine the amount of crude fat present in a sample. The procedure involves weighing 20-g samples into individual thimbles, which were then placed into a pre-programmed Soxtec Oil Extraction Apparatus with specified temperature and recovery time settings. Petroleum ether with a boiling point range of 40–60 °C was used as the solvent for the oil extraction process. Following 6 h of extraction, approximately 90% of the solvent was retrieved, and the remaining solvent is evaporated using an oven. The amount of crude oil extracted was then quantified and expressed as a percentage [26].

#### 2.4.6 Moisture content

For moisture content estimation, 5 g sample was weighed in a previously dried and tared moisture dish. The dish was placed in an air oven maintained at 105 ± 2 °C and dry for 2 h [26]. Cooled in a desiccator and weighed.

$$Moisture\;\left(\%\;weight\right)=\frac{100\;\left(M\;1\;–\;M\;2\right)}{M\;1\;–\;M}$$

Where, M 1 = weight in gm of dish with material before drying M 2 = weight in gm of dish with the dried material M = weight in gm of empty dish.

#### 2.4.7 Total ash

The determination of ash content was carried out following the AOAC method [27]. In this method, 10 g of the sample were weighed out five times into silica crucibles. The crucibles were then placed in a muffle furnace and heated at 600 °C for about 3–5 h. After the heating process, the crucibles were removed from the furnace and allowed to cool in a desiccator before being weighed to determine the weight of the ash produced. To ensure complete ashing, the crucibles were heated again in the furnace for an additional half-hour, cooled, and then weighed again.

$$Ash\;\left(\%\right)=\frac{Weight\;of\;ashed\;sample}{Weight\;of\;sample\;taken}$$

#### 2.4.8 Total carbohydrates

The total carbohydrate content in the mix was determined mathematically as *per cent* dry weight basis [26], as follows:

TotalCarbohydrates(%)=100−(%crudeprotein+%crudefat+%Moisture+%ash)(aspercentofdryweight)

#### 2.4.9 Energy value

The energy value of value-added products was calculated using the following relation by the method given by Rangana [26].

Energy value in kcal = (Crude protein × 4.1) + (Crude fat × 9.3) + (Carbohydrate × 4.1).

#### 2.4.10 Total phenolics

To determine the total phenolic content of the optimized mix, the Folin-Ciocalteu reagent method was used according to the procedure described by Bray and Thorpe [28], with catechol as a standard. One gram of the sample was taken and ground with 10 mL of 80% ethanol in pestle and mortar, and centrifuged for 20 min at 1000 rpm and filtered. The filtrate was evaporated in the oven up to dryness and the dried extract was dissolved in 5 mL distilled water. Next, 0.2–2.0 mL of the extract was taken in separate test tubes and the volume was made up to 3 mL. To each tube, 0.5 mL of Folin-Ciocalteu reagent was added and allowed to react for 3 min. Then, 2 mL of Na_2_CO_3_ (20%) was added to the mixture and thoroughly mixed. The test tubes were placed in a boiling water bath for 1 min and then cooled. The optical density of the sample was measured at 650 nm using a Spectronic-20 spectrophotometer. The concentration was determined according to the standard procedure from the standard curve, which was prepared using different concentrations (8–32 μg/mL) of catechol and the results were expressed as mg *per* 100 g.

#### 2.4.11 Antioxidant activity

The antioxidant activity or free radical scavenging activity was determined using the method described by Brand-Williams et al. [29]. DPPH (2,2-diphenyl-1-picrylhydrazyl) was used as a source of free radicals. A solution of 3.9 mL of 6 × 10^−5^ mol/L DPPH in methanol was added to a cuvette containing 0.1 mg of sample extract and kept in the dark for 30 min. The absorbance was measured at 515 nm against methanol as a blank. The remaining concentration of DPPH was calculated using the following equation:

$$Antioxidant\;activity\;\left(\%\right)=\frac{Ab_{\left(B\right)}\;―\;Ab_{\left(S\right)}}{Ab{\;}_{\left(B\right)}}\times 100$$

here, Ab _(B)_ = Absorbance of blank Ab _(S)_ = Absorbance of sample.

#### 2.4.12 Total plate count

The total plate counts of the instant mix were determined using the standard plate method described by Maturin and Peeler [30]. A 25 mg of the sample was mixed with 225 mL of sterile water, and a 10-fold serial dilution was made up to 10^−5^ dilutions. The dilutions of 10^−4^ and 10^−5^ were surface spread on nutrient agar media, which contains beef extracts, yeast extracts, peptone, and sodium chloride in deionized water. The media plates were then incubated at 37 °C for 24 h, after which the colony-forming units were counted. The results were expressed as CFU/g after 24 h of incubation.

### 2.5 Sensory analysis

A panel of 14 semi-trained judges, consisting of seven males and seven females, conducted the sensory evaluation of the product. The evaluation included the parameters of taste, colour, flavor, texture, and overall acceptability (OAA), which were rated on a 9-point hedonic scale (whereby, 9 = Like extremely; 8 = Like very much; 7 = Like; 6 = Like slightly; 5 = Neither like nor dislike; 4 = Dislike slightly; 3 = Dislike moderately; 2 = Dislike; 1 = Dislike extremely) [31]. Coded samples were provided to the judges in separate chambers, and they were allowed to rinse their mouths with water between each sampling. Further, written consent was obtained from the judges for both the sensory evaluation and research presentation.

### 2.6 Statistical analysis

Canonical correlation analysis (CCA) is a multivariate statistical technique used to determine the relationships between two sets of variables. In this case, CCA was used to determine the effect of independent variables (BGFD and BGPD) on the quality parameters (taste, colour, flavour, texture, and OAA). The software calculates eigenvalues, which indicate the level of variance, and the variance in percent is manually calculated by dividing each set of eigenvalues by the total number of eigenvalues. The set of canonical correlation with the maximum variance was selected for the study, and another were rejected [31]. Principal component analysis (PCA) is a technique used to reduce the dimensionality of a dataset, while retaining as much of the original variability as possible. In this study, PCA was used to analyze the relationship between BGFD and BGPD in different runs for sensory parameters using Minitab Statistical Software (Version 18.0, Minitab Inc.). The results of the optimized recipe were expressed as mean values ± standard deviations in Excel (MS office version 2016). Each analysis assay was done five times from the same sample to determine reproducibility.

## 3 Results and discussion

### 3.1 RSM Central Composite Design (CCD)

#### 3.1.1 Taste

**Fig. 1:**
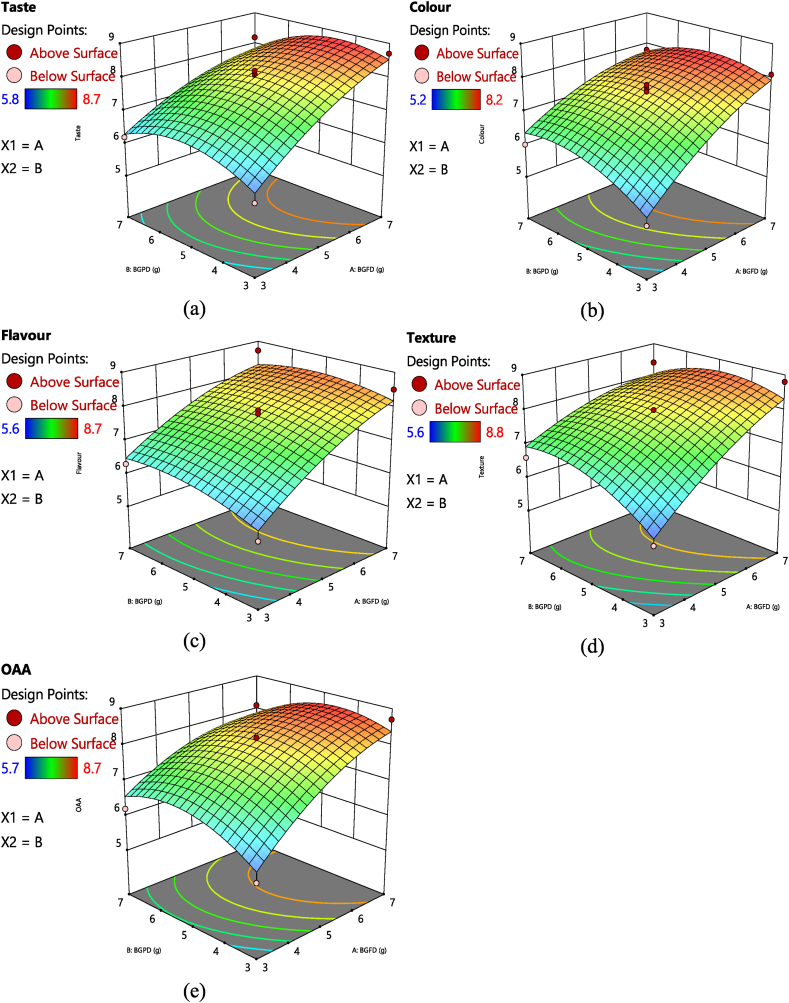
Effect of BGPD and BGFD on (a) taste, (b) colour, (c) flavour, (d) texture, (e) OAA of instant bottle gourd *kheer* mix. (For interpretation of the references to colour in this figure legend, the reader is referred to the Web version of this article.)

The evaluation of taste plays a crucial role in organoleptic assessment. Through the RSM CCD method, various runs of BGFD & BGPD were explored to determine their impact on the taste parameter, yielding a range of 5.8–8.7 (Table 1). The significant F-value (26.90) of the quadratic model confirms its significance in relation to taste. The linear level addition of BGFD in the bottle gourd *kheer* mix demonstrated a notable influence on taste. Additionally, Table (2) indicates that both BGFD and BGPD exhibit significant effects at the quadratic level (p < 0.05). Fig. 1(a) further illustrates that increasing the quantity of BGFD in the mix directly corresponds to an incremental improvement in taste. On the other hand, augmenting BGPD up to a certain threshold enhances taste, but surpassing that threshold leads to a decline in taste. The regression equation (*R*^*2*^), which accounts for 95% of the total taste variation, signifies the model's high significance in predicting taste. Several aliphatic aldehydes, including octanal, nonanal, and decanal, exhibit potent fruity, citrusy, and floral aromas within the bottle gourd [32]. These compounds potentially contribute to an improved taste experience in the final product due to their higher content of BGFD. Furthermore, the observation that the increment in taste is attributed to the presence of minerals and freshness in the bottle gourd kheer mix is noteworthy, as it suggests that the addition of natural ingredients can enhance the taste of the product. Similar improved sensory scores were obtained for pumpkin *kheer* mix by Adil et al. [33].

**Table 1:** Effect of treatments on organoleptic quality of optimized instant bottle gourd *kheer* mix.

Treatments	Process variables		Responses				
	BGFD (g)	BGPD (g)	Taste	Colour	Flavour	Texture	OAA
**T1**	5	5	8	7.8	7.9	8	8.2
**T2**	7	3	8.7	8.1	8.5	8.8	8.7
**T3**	5	5	7.8	7.6	7.7	8	8.2
**T4**	3	7	6.2	6	6.3	6.6	6.2
**T5**	3	3	5.8	5.2	5.6	5.6	5.7
**T6**	7	7	8.2	7.8	8.7	8.4	8.1
**T7**	5	5	8.1	7.7	7.7	8	8.2
**T8**	5	5	8	7.4	7.8	8	8.1
**T9**	5	5	8.2	7.5	7.6	7.9	8.1
**T10**	7.8	5	8.5	8.2	8.1	8.2	8.2
**T11**	2.2	5	6.3	6	6.1	6.6	6.5
**T12**	5	7.8	6.6	7.2	7.3	7.4	7.1
**T13**	5	2.2	7.2	6.5	7	6.6	7
**T14**	5	5	8	7.6	7.7	8	8

*Fixed variables: Milk powder- 50 g; sugar 15 g, small cardamom powder-1g.

#### 3.1.2 Colour

Table 1, employing a hedonic scale, revealed that the colour value of the bottle gourd *kheer* mix ranged from 5.2 to 8.2. The addition of BGFD at the linear level, as well as BGFD and BGPD at the quadratic level, significantly impacted the colour value of the mix (p ≤ 0.05). The regression equation (*R*^*2*^) in Table 2 accounted for 95% of the total colour variation in the mix. Importantly, Fig. 1(b) visually displayed that incorporating BGFD in the range of 3–7 g exerted a significant positive influence on the colour value of the bottle gourd kheer mix. These findings align with a previous study conducted by Barot et al. [34], which reported an enhancement in the colour and appearance value of ice cream by incorporating bottle gourd puree ranging from 1.17% to 6.8%. This supports the current study's observations that the addition of fresh bottle gourd shreds effectively enhances the colour value according to the panelists, both at the linear and quadratic levels. Additionally, pre-treatment blanching may have helped to maintain the colour and retention during the *kheer* mix preparation, which is consistent with a previous study that reported similar results on the colour value when using ohmic and conventional blanching techniques in bottle gourd [35].

**Table 2:** Analysis of variance, regression analysis, fractional factorial design for F-value to the preparation instant bottle gourd *kheer* mix.

Variables	df	Estimated coefficient					F-values				
		Taste	Colour	Flavour	Texture	OAA	Taste	Colour	Flavour	Texture	OAA
**Model**	5	10.94	10.07	9.52	9.34	10.13	26.90	26.98	15.78	13.20	17.42
**A-BGFD**	1	1	0.98	1.02	0.91	0.91	98.60*	102.15*	68.46*	46.61*	57.34*
**B-BGPD**	1	−0.12	0.19	0.16	0.22	0.01	1.38	3.72	1.82	2.65	0.01
**AB**	1	−0.22	−0.27	−0.13	−0.35	−0.27	2.49	4.05	0.52	3.46	2.60
**A**^**2**^	1	−0.29	−0.30	−0.28	−0.25	−0.40	7.61*	8.90*	4.77	3.37	10.05*
**B**^**2**^	1	−0.54	−0.42	−0.25	−0.45	−0.55	26.42*	17.86*	3.95	10.77*	19.06*
**Lack of fit**	3										
**R**^**2**^		0.95	0.95	0.92	0.90	0.93					
**Adj R**^**2**^		0.91	0.91	0.86	0.84	0.87					
**C·V. %**		3.78	3.80	4.68	4.96	4.49					

#### 3.1.3 Flavour

Table 1 showed that the flavour value, another critical sensory parameter, ranged from 5.6 to 8.7 across different runs. Table 2 revealed that only BGFD alone significantly influenced the flavour content of the bottle gourd *kheer* mix at a linear level. Fig. 1(c) visually depicted a positive correlation between the addition of BGFD and the flavor intensity. The regression equation (*R*^*2*^) accounted for 92% of the total variation in flavour content. Adding milk powder improved the flavour, which is consistent with the findings of Bakshi et al. [36] and supports the present study's results. Furthermore, in a study on bottle gourd burfi, increasing the bottle gourd pulp concentration enhanced all organoleptic properties, including flavour, texture, colour, and overall acceptability [37]. Similarly, in the present study, the addition of BGFD enhanced the flavour content in the bottle gourd *kheer* mix. The results revealed that the highest scores for appearance, colour, texture, taste and flavour were obtained by B2 (65%; range 8.5–8.7; overall acceptability score of 8.58), being liked very much, followed by B1 (50%; overall acceptability score of 8.3) in a previous study conducted by Priya et al. [38].

#### 3.1.4 Texture

The texture content of the prepared bottle gourd *kheer* mix ranged from 5.6 to 8.8, as shown in Table 1. The addition of BGFD at the linear level and BGPD at the quadratic level significantly influenced the texture content (p < 0.05), as indicated in Table 2. Fig. 1(d) also supports this finding, showing that the texture of the bottle gourd kheer mix improved with the addition of BGFD from 3 g to 7 g. The high dietary fiber content of bottle gourd, which is approximately 24.14 g/100 g on dry matter basis [39], might be responsible for the texture in the bottle gourd *kheer* mix. The addition of BGPD might have further increased the textural properties of the mix. Collar et al. [40] suggested that dietary fiber can modify the consistency and texture quality of a product. Similar results were observed when wheat bran was added to bread [41], potato fiber was added to bakery products [42], and pear cactus fiber was added to bakery products [43]. The microstructure of fibers plays a crucial role in increasing the holding capacity [42,44,45]. The size and source of fiber can also influence textural quality [46,47]. Therefore, the texture difference observed in the bottle gourd *kheer* mix can be attributed to the different combinations used in the study.

#### 3.1.5 OAA

Similar to taste and colour, the addition of BGFD at the linear level, while, BGFD and BGPD at the quadratic level significantly influenced the overall acceptability (OAA) of the bottle gourd kheer mix (Table 1 & Fig. 1(e)). The OAA scores ranged from 5.7 to 8.7 on the 9-point hedonic scale (Table 1). The regression equation (*R*^*2*^) accounted for 93% of the total variation in the OAA of the bottle gourd kheer mix. The findings of a previous study by Priya et al. [38] align with the current results, indicating that bottle gourd *kheer* mixes containing 65% khoa (a type of milk solid) received the highest scores for appearance, colour, texture, taste, and flavor. These mixes garnered an overall acceptability score of 8.58, indicating strong preference among the participants. The similarity between these values and the findings of the present study highlights the consistency and reliability of the observations regarding the favorable attributes of the bottle gourd *kheer* mixes. According to a study conducted by Shelke et al. [48], it was discovered that bottle gourd kheer containing 20% pulped bottle gourd and 80% cow milk received the highest sensory scores. This finding aligns with the current study, suggesting that increasing the quantity of fresh bottle gourd positively impacts the sensory attributes of the kheer.

### 3.2 Numerical optimization

The software analysis recommended an optimal combination of BGFD and BGPD at 7.0 g and 3.0 g, respectively, for the development of bottle gourd kheer mix, which resulted in a desirability of 1.00. Table 3 provides a detailed overview of the optimized process parameters and their impact on the quality attributes of the product.

**Table 3:** Numerically optimized conditions for the preparation of instant bottle gourd *kheer* mix.

Process variables			Responses					
**BGFD (g)**	**BGPD (g)**		Taste	Colour	Flavour	Texture	OAA	Desirability
7.0	3.0	Predicted (Through RSM)	8.5	7.9	8.2	8.3	8.4	1.0
		Experimental	8.9	7.7	7.8	8	8.2	
		Differences	−0.4	0.2	0.4	0.3	0.2	

*Milk powder (50.0 g), Table sugar (15.0 g) & Small cardamom powder (1.0 g).

### 3.3 Principal Component Analysis (PCA)

**Fig. 2:**
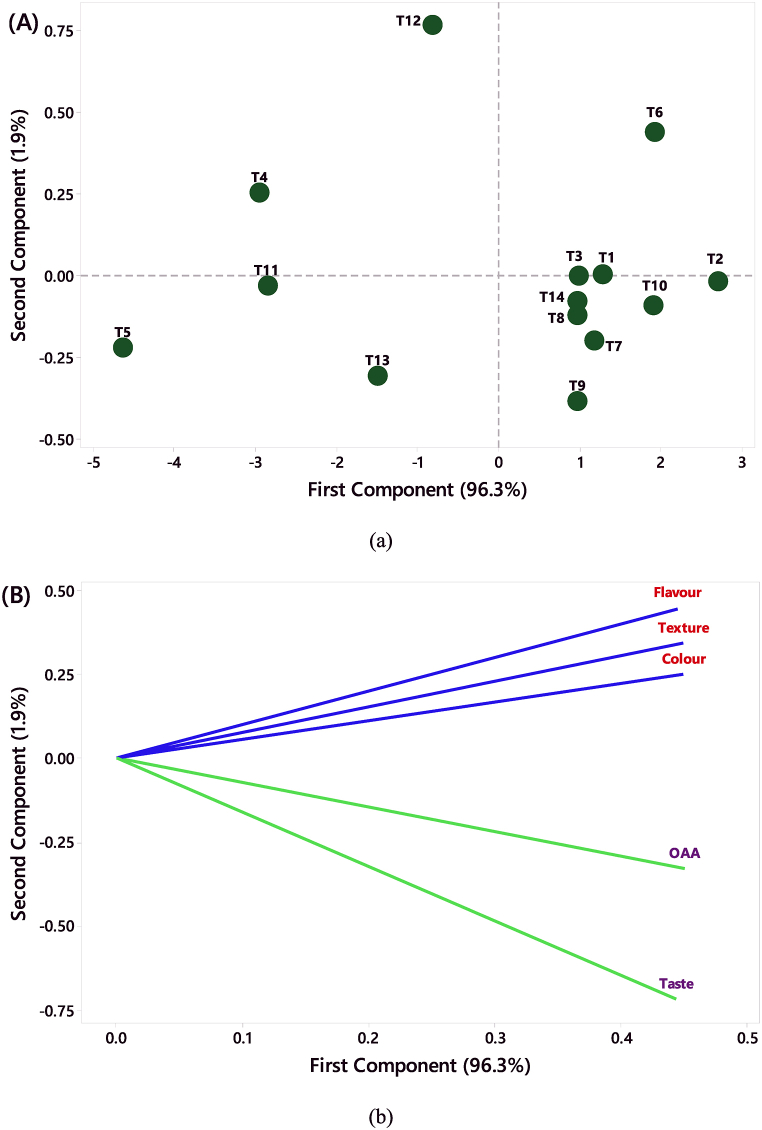
Principle component analysis (PCA) of instant bottle gourd *kheer* mix (a) the combinations of different treatments, (b) the different sensory parameters.

A PCA model was developed using different treatments obtained from the CCD RSM runs to distinguish similar and different treatments based on organoleptic scores. The sum of Principal components PC1 and PC2 explained a very high variability of 98.2% among the treatments (Fig. 2(a)). The PC1 and PC2 account for 96.3% and 1.9% of the total variance among the treatments for organoleptic characteristics *i.e.* taste, colour, flavour, texture and OAA. Since the PC1 is the component that identifies the effect of treatments on the sensory parameters, it accounts for the largest variation [49]. Treatments plotted in quadrants I and IV had better sensory properties as they were closer to the vectors, and treatment T_2_ was the most acceptable. The treatments in quadrants II and III had poor organoleptic scores and the treatment T_5_ was the least liked. Treatments T_1_, T_2_, T_3_, T_6_, T_7_, T_8_, T_9_, T_10_ & T_14_ are considered in the group with better organoleptic properties and had a major share of BGFD. The treatments T_4_, T_5_, T_11_, T_12_ and T_13_ were grouped to the least liked category and had the major share of BGPD in the treatment combinations. The PC1 accounts for 96.3% of the variability (Fig. 2(b)) and it was positively loaded by taste (0.443), colour (0.449), flavour (0.445), texture (0.449) and OAA (0.450), while, PC2 accounts for 1.9% of the variability and it was positively loaded by colour (0.250), flavour (0.446) and texture (0.343) while least affected for OAA (−0.328) and taste (−0.717). PCA is a useful tool for studying consumer behavior towards instant products [50], and in this study, it was applied to evaluate the preference for instant *kheer* mix based on individual liking.

### 3.4 Canonical correlation analysis (CCA)

**Fig. 3:**
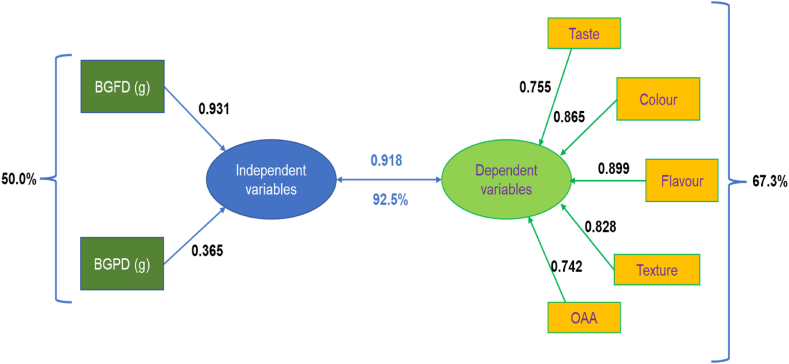
Canonical correlation of independent variables (BGFD & BGPD) on sensory parameters of instant bottle gourd *kheer* mix.

According to the results of canonical correlation analysis, the combined variation of BGFD and BGPD on sensory parameters, such as taste, colour, flavor, texture, and OAA, accounted for 92.5% (Table 4). The independent canonical loadings for BGFD and BGPD were 0.931 and 0.365, respectively (Fig. 3). The total variance of BGFD and BGPD on the responses is 50%. The canonical loading values obtained for the responses *i.e.* taste, colour, flavour, texture and OAA were 0.755, 0.865, 0.899, 0.828 and 0.742, respectively. The variance among these responses on the dependent variables was high *i.e.* 67.3% (Table 3). Moreover, Fig. 3 shows that the loading value (0.931) for BGFD is higher than that of BGPD, indicating that BGFD has a greater influence on the dependent variable than BGPD. Additionally, an increase in BGFD is likely to have a more significant positive effect on flavor compared to other variables, while its effect on OAA was least effected. It is worth noting that a previous study on spinach-lemon-tulsi beverage development also employed CCA to investigate the relationship between independent variables and individual dependent variables [31].

**Table 4:** Functional properties of optimized bottle gourd *kheer* mix.

Parameters	*Kheer* mix
TSS (°B)	27.00 ± 0.20
Reducing sugars (%)	10.79 ± 0.14
Total sugars (%)	16.75 ± 0.36
Titratable acidity (%)	0.896 ± 0.120
Crude Proteins (%)	10.76 ± 0.82
Crude fat (%)	7.63 ± 0.22
Moisture content (%)	7.9 ± 0.3
Ash (%)	1.50 ± 0.02
Carbohydrates (%)	72.21 ± 0.42
Energy (kcal)	400.55 ± 0.28
Antioxidant activity (%, DPPH)	46.00 ± 0.34
Total phenols (mg/100 g)	4.99 ± 0.62
TPC (CFU/g)	4.3 × 10^6^

### 3.5 Functional properties of optimized instant bottle gourd kheer mix

Table 4 presents the functional properties of the standardized recipe for bottle gourd kheer mix, which includes BGFD 4.25 g, BGPD 4.78 g, milk powder 50 g, sugar 15 g and small cardamom 1 g. The mix was found to have a TSS of 27 °B, reducing sugars of 10.79%, total sugars of 16.75%, and titratable acidity of 0.896%. In terms of macronutrient content, the mix had a good protein content of 10.76%, crude fat of 7.63%, ash content of 1.5%, carbohydrates of 72.21%, and energy content of 400.55 kcal. Furthermore, the moisture content and total phenols were 7.9% and 4.99 mg/100 g, respectively. The antioxidant activity was 46% as DPPH, while TPC in the optimized bottle gourd kheer mix was 4.3 × 10^6^. In a previous study, higher antioxidant activity (around 83–85% as DPPH) was observed in blanched bottle gourds [35]. Kaddumukasa et al. [51] conducted a study on the microbial load of unpasteurized fruit juices, which is at par with the values obtained in the present study.

## 4 Conclusion

A bottle gourd kheer mix with higher sensorial acceptability can be prepared using 7 g BGFD and 3 g BGPD mixed with 50 g milk powder, 15 g sugar, and 1 g small cardamom powder. The mix was also having high nutritional and phytochemical characteristics and hence can be recommended as a health food. The short cooking time of the product also make it a convenient food for working class. This product has a vast potential to commercialized. Pomace, being an unutilized by-product of vegetable processing industry possesses environmental hazard and the prepared product can also solve the environmental issue to some extent. This can also help to earn the extra income to the processors.

## Author contribution statement

Ghan Shyam Abrol: Conceived and designed the experiments; Performed the experiments; Analyzed and interpreted the data; Wrote the paper. Amit Kumar Singh, Ranjit Pal: Performed the experiments; Analyzed and interpreted the data; Contributed reagents, materials, analysis tools or data. Ashwani Kumar: Analyzed and interpreted the data; Wrote the paper. Priyanka Sharma, Gaurav Sharma: Contributed reagents, materials, analysis tools or data.

## Data availability statement

Data included in article/supp. material/referenced in article.

## Ethics statemen

The sensory evaluation studies were conducted according to established ethical guidelines, and informed consent obtained from the participants.

## Declaration of competing interest

The authors declare that they have no known competing financial interests or personal relationships that could have appeared to influence the work reported in this paper.

## Appendix A Supplementary data

SENSORY PROFORMA.doc SENSORY PROFORMA.doc
