# Supplementary material for: Utilization of bottle gourd (Lagenaria siceraria (Mol.) Standl.) pomace for the preparation of instant kheer (dessert) mix
Source: Heliyon. 2023 Jul 29;9(8):e18533. doi: 10.1016/j.heliyon.2023.e18533 (PMC10415662; doi:10.1016/j.heliyon.2023.e18533)
Supplement: SENSORY PROFORMA.doc [file mmc1.doc]

**SENSORY PROFORMA**

**You have provided the coded samples of instant bottle gourd *kheer* mix, kindly evaluate it on the basis of your liking**

Evaluator Name: _________________________________________

Date of Evaluation: _________________________________________

| Coded Sample | Taste | Colour | Flavour | Texture | Overall Acceptability |
| --- | --- | --- | --- | --- | --- |
| 221 |  |  |  |  |  |
| 232 |  |  |  |  |  |
| 243 |  |  |  |  |  |
| 254 |  |  |  |  |  |
| 256 |  |  |  |  |  |
| 258 |  |  |  |  |  |
| 265 |  |  |  |  |  |
| 277 |  |  |  |  |  |
| 282 |  |  |  |  |  |
| 284 |  |  |  |  |  |
| 289 |  |  |  |  |  |
| 290 |  |  |  |  |  |
| 293 |  |  |  |  |  |
| 298 |  |  |  |  |  |

**Scale for 9- point Hedonic ratings**

9 : Like extremely 4 : Dislike slightly

8 : Like very much 3 : Dislike moderately

7 : Like moderately 2 : Dislike very much

6 : Like slightly 1 : Dislike extremely

5 : Neither like nor dislike

- - Special observations, if any with particular treatment

__________________________________________________________________________________________________________________

**Signature of evaluator**
